# Personal Resources of Winter and Summer Hikers Visiting the Tatra National Park, Poland

**DOI:** 10.3390/ijerph19073810

**Published:** 2022-03-23

**Authors:** Piotr Próchniak, Agnieszka Próchniak

**Affiliations:** 1 Institute of Psychology, University of Szczecin, 71-017 Szczecin, Poland; 2 Department of Sociology, Pomeranian University, 76-200 Slupsk, Poland; agnieszka.prochniak@apsl.edu.pl

**Keywords:** personal resources, mountain hikers, weather, core self-evaluations, psychological capital

## Abstract

To assess personal resources: Core Self Evaluations and Psychological Capital (HERO) of 95 winter Hikers (*M* age = 27.10 yrs.; *SD* = 7.30) and 98 summer Hikers (*M* age = 25.30 yrs.; *SD* = 5.40) visiting the Tatra National Park (Poland). The hikers filled in seven scales. These were: the *Delta Questionnaire for measure Locus of Control*, the *Neuroticism scale* (from the NEO-FFI), the *Rosenberg Self-Esteem Scale (SES)*, the *Hope Scale,* The *Generalized Self-Efficacy Scale (GSES)*, the *Ego Resiliency Scale*, and The *Life Orientation Test Revised (LOT-R).* The results indicated significant differences between winter and summer hikers in the Tatras. The winter hikers scored higher on self-esteem, hope, self-efficacy, resilience, and optimism, and lower on external locus of control and neuroticism than summer hikers. This study also examined the factor structure of the personal resources in the hikers’ samples. The results suggested that the scales extracted two factors: Cognitive Resources and Affective Reactivity. These factors discriminate between winter and summer hikers. The Cognitive Resources factor is more important in effective adaptation to the wild world of nature than Affective Reactivity.

## 1. Introduction

Hiking is an outdoor activity which consists of walking on a trail in different landscapes. Hiking is increasingly popular. Every year, more and more people go on foot through mountains, hills, forests, beaches, or other natural environments. The positive consequences of hiking are multifaceted: feeling healthy, feeling relaxed, improving cognitive skills, or deeply experiencing nature. Hiking tests one′s endurance and psychological capacity. However, we can observe hazards occur from time to time which can overshadow the benefits of the hiking. Hazards in hiking are connected with injuries from falls on the trail, injuries caused by animals (e.g., insects or snakes), and injuries from inclement weather, such as hypothermia and heat exhaustion. Hikers can be injured by forces of nature such as veld fires and lightning. Finally, hikers can lose the trail [1,2].

Sometimes hiking is used interchangeably with other words. For example, trekking describes multi-day hiking in the mountainous regions of Asia or South America. Nordic walking is hiking with specially designed walking poles. Bushwalking is hiking through the bush in Australia. In the United States multi-day hikes are referred to as backpacking. In New Zealand hiking for at least one overnight stay is known as tramping. Through-hiking means walking an established long-distance trail end-to-end within one hiking season. We have also dog hiking or glacier hiking [3,4,5].

One of the most popular types of hiking is mountain hiking [6]. Mountain hiking is the activity of going for long walks in mountainous areas with altitude differences [3], (p. 1). Every year, more and more people set off on a hiking in different mountains around the world. Mountain hiking is physically demanding and does require personal resources. Surprisingly though, personal resources of mountain hikers have rarely been analyzed in environmental research. Thus, the purpose of this investigation is to analyze personal resources of hikers visiting the Tatra National Park in the summer and in the winter.

### 1.1. Hiking in Tatra Mountains

The Tatra Mountains are the highest mountain range in Carpathian Mountains, which are the second longest mountain system in Europe. The Tatras are located in Poland and Slovakia, encompassing the area of 19°45′36″ E and 20°09′00″ E, as well as 49°10′42″ N and 49°20′05″ N. They are alpine-type mountains (the highest peak is Gerlach, 2655 m above sea level). The zone of bare rock occurs in the Tatras at an altitude higher than 2300m above sea level. The lower ranges of Tatra Mountains are the natural home of the amazing flora (e.g., vascular plants, fungi, mosses, or slime molds) and fauna (e.g., bears, Tatra chamois, wolf, red deer, Alpine marmot, and two hundred of species of bird). About seventy five percent of the Tatras is covered by forests [7,8,9].

The Tatra Mountains are protected by two national parks, namely the Tatrzański Park Narodowy (TPN) in Poland and Tatranský Národný Park (TANAP) in Slovakia.

In the Tatras, hikers can find a network of hiking trials in different levels of difficulty. Levels of difficulty vary based on seasonal weather conditions. Summer is a relatively cool season for hiking in the Tatras. During the summer months, average day temperatures usually fall between 20 °C (68 °F) and 25 °C (77 °F). There is no snow or ice on the trails. Stronger wind occurs only on mountain ridges and peaks. Rain is intensive for short periods of time, usually 30 min to a few hours. On very hot days, rain is accompanied with thunderstorms. Morning fog is a common condition in the summer period, but the trails are very well marked [10]. Hiking in the summertime in the Tatras does not require any special equipment (e.g., helmet, crampons, axes, clip hooks, ropes, etc.), but hikers must be in good physical condition.

The main risks in summer hiking relate to thunderstorms, possibilities of falls, or attacks by animals (but, in fact, attacks by, e.g., bears on hikers in the Tatras are extremely rare). Summer hikers are driven by relaxation, discovering, and experiencing new environments in relatively safe surroundings [11,12].

Hiking conditions in winter change dramatically. Heavy snow and ice on the trail are very common. Snowfall limits visibility, erases tracks, and covers trail markings. There is also a large risk of avalanches. The average temperature in the winter season is below 0 °C (32 °F). Extreme temperatures can go down to −40 °C (−40 °F). High winds are characteristic for the winter season in the Tatras [10].

Winter hiking in the Tatras usually requires more skills, competence, or attention to detail because there are more risks on route, such as avalanches, snow, intense cold and ice, or real possibilities of falls from great heights into the abyss. Winter days are much shorter than in the summer. Very often, hikers must have special equipment, such as an avalanche transceiver, probes, helmet, or ice axe. They must manage stress, pain, or injury and, thus, they need personal resources to deal with risk in winter hiking [11,13,14].

### 1.2. Personal Resources

The word “personal” means that individual properties can function as a way of dealing with the outside world [15]. In turn, the word “resources” means resources that can be used in stressful situations. To Pearlin and Schooler, personal resources are “personality characteristics that people draw upon to help them withstand threats posed by events and objects in their environment” [16], (p. 5). To Hobfoll, personal resources are defined as things that one values; specifically, objects, states, and conditions. People strive to maintain their current resources and pursue new resources. This author also claims that people have to invest resources, which can be personal, to protect themselves against resource loss, and to gain resource. Loss resources are disproportionately more salient than gain resources [17].

Personal resources allow people to achieve individual goals, reducing physiological and psychological costs associated with demanding conditions of surroundings and stimulating personal growth. Personal resources promote subjective well-being [18,19]. Ideas of personal resources can be found in different conceptions; for example, Core Self-Evaluations [20] and Psychological Capital [21].

Surprisingly though, wilderness adventure has rarely been studied in the context of personal resources. Researchers rather analyzed the motivational aspects of outdoor adventure: goal achievement, sensation seeking, escape from boredom, pushing personal boundaries, and overcoming fear [22,23]. They also focused on several possible psychological, social, physical, or even spiritual benefits of activity in extreme environment. These results indicate that extreme recreationists talk about a sense of freedom, a full sense of their lives, or a sense of connection with nature [24,25]. They experience deep satisfaction with life more often than anxiety, boredom, or apathy [26]. Adventure in extreme environment is a source of exciting positive emotions and deep satisfaction [27].

The most common resource research among adventurous persons concerned rather single variables, for example, self-efficacy or neuroticism [28,29]. Surprisingly, we know almost nothing about structure of personal resources or profiles of personal resources recreationists in wilderness. Therefore, in this article, personal resources of hikers will be analyzed in a more comprehensive way. In the first phase, some theories of personal resources will be presented and, following this, there is an analysis of personal resources among winter and summer hikers using some theories of personal resources.

#### 1.2.1. Core Self-Evaluations

The concept of core self-evaluations was first introduced by Judge, Locke, and Durham [20]. This construct preliminary was developed as a dispositional predictor of job satisfaction, but has been expanded to predict a variety of other outcomes.

Core self-evaluations include four personality dimensions: locus of control, neuroticism, self-efficacy, and self-esteem. These resources appear to play a key role in adaptation to the wild nature.

The locus of control describes the tendency to attribute life′s events to their own doing. People evaluate possibilities of action differently depending on whether they feel that, in a given situation, the outcomes depend on themselves, their own abilities or effort, or whether the believe that events are contingent upon chance. A selection of given courses of action will be different depending on whether one believes in the effectiveness of one’s behavior (internal locus of control) or does not (external locus of control) [20].

Neuroticism is defined as an enduring tendency to experience unpleasant emotions easily. Neurotics display high sensibility, emotional instability, and low perseverance. They show little energy and tend to feel unhappy. Threat and anxiety induce them to react defensively by avoiding risk [30].

Self-efficacy is ‘‘belief in one’s capabilities to organize and execute the courses of action required to produce given attainments” [31], (p. 3). Self-efficacy beliefs determine whether individuals are optimists or pessimists in new and risky situations. Furthermore, self-efficacy influences the way people motivate themselves in achieving their goals. Individuals undertake challenges where self-efficacy is high and avoid risky goals or tasks where self-efficacy is low [31].

Finally, self-esteem is characterized by one’s global self-regard and the extent to which she/he accepts herself/himself. Self-esteem includes beliefs about oneself as well as emotional states. Individuals with high self-esteem increase their efforts and persistence in the face of risk or potential failure. Thus, high levels of self-esteem sustain motivation. Self-esteem plays a key role in well-being [32].

Only some of the constructs of Core Self-Evaluations have been studied in a mountain context. For example, one study indicates that a high self-efficacy is related to free choice of climbing and to the difficulty of doing outdoor climbing [33]. Climbers with high self-efficacy can judge themselves as capable of coping with stress and they can set themselves challenging goals in the wilderness and maintain a strong commitment to them. Those who are self-efficacious may engage more easily in extreme activities, in spite of adversity [29].

Different studies indicate that climbers have lower scores on neuroticism than controls. For example, Egan and Stelmack [34] tested traits of personality among climbers during expedition on K2 peak in the summer. Climbers displayed lower scores on neuroticism (as well as extraversion and psychoticism) compared to the controls. In the study by Levenson [35], rock climbers had lower scores on anxiety traits than norms (Robinson, 1985). Similar results were found by Tok [36].

Other components of the Core Self-Evaluations have been rarely considered in mountaineering. Saeid Bahaeloo-Horeh and Shervin Assari tested the impact of mountaineering program on self-esteem. The participants completed The Rosenberg Self-Esteem Scale (scale for diagnosing self-esteem) before and after mountaineering. Participation in a single mountaineering program improved climbers’ sense of self-esteem [37].

#### 1.2.2. Psychological Capital (HERO)

Psychological Capital is defined as examining the processes by which positive attitudes, feedback, and criticism contribute to the functioning and development of an individual, group, or corporation [21]. The four fundamental characteristics of Psychological Capital (hope, self-efficacy, resiliency, and optimism) are the key factors needed to form a psychological capital structure.

Hope is defined as a positive motivational state where two basic elements, successful feelings of agency (or goal oriented determination) and pathways (or proactively planning to achieve those goals), interact. Hope is a strong predictor of the lack of depressive symptoms, positive and negative affectivity, quality of friendship, and health as indicators of optimal functioning in one’s private and professional life [38].

Self-efficacy is defined as a person’s confidence in their ability to achieve a specific goal in a specific situation [39]. This construct was described in the previous section.

The concept of resilience is taken from psychiatric literature, but it is actually defined as an ordinary characteristic of normal development. Resilience is defined as an ability to recuperate from stress, conflict, failure, change, or increase in responsibility. In a broader sense resilience can be defined as a dynamic process which reflects relatively good individual adaptation, in spite of dangers or traumatic experiences that one endures. Resilience moderates the relationship between stressful events and illness. It is a trait which is developed throughout one’s life as a result of various experiences [40].

Optimism refers to one’s perspective on future personal and social events, in which there will be an abundance of good things and a scarcity of bad things. People with high optimism experience positive emotions, even amidst stress. They are determined and engaged in more adaptive coping strategies and less maladaptive coping than those who perceive themselves as low-optimistic individuals [41].

Hope and optimism weren’t studied in a climbing context. The role of self-efficacy in outdoor adventure was described in the previous section. Studies of resilience have rarely been considered in mountaineering. Tukaiev et al. tested resilience among a group of 60 Ukrainian extreme climbers. The results indicate that extreme climbers had higher scores on resilience compared to athletes practicing non extreme sports [42]. In a recent study, psychological resilience was investigated “live” (e.g., in the moment) in challenge team members involved in a 25-day extreme endurance challenge. The results of the study highlighted the individualized, complex, and dynamic nature of psychological resilience within extreme environments [43].

As we said, personal resources of adventure participants weren’t analyzed in a more comprehensive way. The most common resource research among adventurous persons concerned rather single variables. Thus, in the current study, winter and summer hikers visiting the National Tatra Park were compared on different personal resources using theories of Core Self-Evaluations and Psychological Capital. Based on the previous studies, it was hypothesized that winter hikers would score higher on personal resources than a group engaging in summer hiking.

## 2. Method

### 2.1. Participants

The total sample included two groups who hiked in Tatra Mountains. The first group of 95 winter hikers who voluntarily participated were all men (*M* age = 27.10 yrs.; *SD* = 7.30). Most of them (65 respondents) lived in cities, and the others (30 respondents) lived in the countryside. All participants had at least a secondary level of education.

They have hiked the Tatra Mountains (Poland) in winter season. All of them hike the mountains during the winter and risk being killed by very difficult mountain conditions. They use specialist equipment: axes and crampons.

The second group was of 98 men who practiced hiking in the summertime in the Tatras (*M* age = 25.30 yrs.; *SD* = 5.40). Most of them (63 respondents) lived in cities, and the others (35 respondents) lived in the countryside. All participants had at least a secondary level of education.

Mountain athletes in this group have personal experiences on relatively safe hiking trails without snow and well-marked routes in the summer period. Hikers in this group had no personal experiences in hiking during the wintertime in the Tatra Mountains.

### 2.2. Procedure

All subjects gave their informed consent for inclusion before they participated in the study. The study was conducted in accordance with the Declaration of Helsinki, and the protocol was approved by the University of Szczecin Institutional Review Board before we began recruiting participants (KB 14/2019). We made contact with hikers during their stays in mountain huts in the summer and the winter periods. Winter hikers were selected on following criteria: they hiked in the winter, and they used axes and crampons; they had more than three years winter hiking experiences; and they were men.

Summer hikers were selected on following criteria: they hiked in summer period; they not used special mountain equipment during exploration of Tatras; they had a few personal experiences in summer hiking (more than three years); they hadn’t personal experiences in winter hiking; and they were men.

Summer and winter hikers completed questionnaires during their stays in Polish mountain huts (Roztoka Hut, Murowaniec Hut, Hut in the Five Polish Lakes Valley, Kondratowa Hut, and Ornak Hut in the Tatra National Park). Participation was voluntary. Hikers completed the questionnaire anonymously.

### 2.3. Measures

#### 2.3.1. Delta Questionnaire

The Delta Questionnaire measures locus of control (LOC) [44]. This questionnaire consists of 24 statements, 14 of which refer to the locus of control (LOC), whereas the other 10 statements make a control lie scale. High scores on the LOC scale indicate external locus of control (*Cronbach’s α =* 0.76).

#### 2.3.2. Neuroticism

Neuroticism scale (from the NEO-FFI) [30] was administered in its Polish adaptation [45]. There are 12-item scales constructed to assess individual differences in neuroticism (*Cronbach’s α =* 0.81).

#### 2.3.3. The Generalized Self-Efficacy Scale (GSES)

The GSES consists of 10 statements, included in one factor [46]. It measures the strength of an individual’s general self-efficacy beliefs in the face of difficult situations and obstacles (*Cronbach’s α* = 0.85). The Polish adaptation was made by Juczyński [47].

#### 2.3.4. Rosenberg Self-Esteem Scale (SES)

The SES is a 10-item scale which consists of five positive and five negative statements [48]. The SES measures Self-Esteem. Coefficient alpha reliability in the Polish version for the SES test was *Cronbach’s α =* 0.77 [49].

#### 2.3.5. The Hope Scale

The Hope Scale is 12-item tool that measures level of hope [50,51]. It consists of two subscales, agency (measures one’s goal-directed energy to pursue one’s goals) (*Cronbach’s α* = 0.82) and pathway (measures one’s extent of creating ways to achieve one’s goal) (*Cronbach’s α* = 0.72). These subscales are highly correlated. In this study the hope construct was measured as means of these subscales.

#### 2.3.6. Ego Resiliency Scale

Ego Resiliency Scale was developed by Block and Kremen [52]. Kaczmarek translated this scale into Polish [53]. Ego Resiliency Scale consists of 14 items. The scale has a satisfactory internal consistency of *Cronbach’s α* = 0.78.

#### 2.3.7. The Life Orientation Test Revised (LOT-R)

The Life Orientation Test Revised (LOT-R) is a 10-item unidimensional scale that was constructed to assess individual differences in generalized optimism. Coefficient alpha reliability in the polish version for the Life Orientation test was *Cronbach’s α* = 0.73 [47].

## 3. Results

The winter and summer hikers were compared on each measure using the Student *t* test (See Table 1).

The winter hikers’ group had a significantly higher mean on the self-efficacy, the self-esteem, the hope, the optimism, and the resiliency than the summer hikers’ group, but lower means on the locus of control and the neuroticism than the summer hikers’ mountain group (*p* < 0.01). Self-efficacy is the factor that most strongly differs betweenboth groups of hikers.

In the next step, a factor analysis was conducted for the scales. The maximum-likelihood method of parameter estimation was chosen [54]. The KMO index was found to be 0.810. Additionally, BTS reached statistical significance *χ2*(55) = 1304.935, *p* < 0.01. The KMO and BTS results indicated that data satisfied the psychometric criteria for factor analysis to be performed. Exploratory factor analysis using the maximum-likelihood method of parameter estimation indicated a two-factor solution upon observing the scree plot (see Table 2).

Additionally, in determining the optimal number of factors to extract, Parallel Analysis (PA) was used [55]. The parallel analysis also showed a strong two-factor solution.

The two factors accounted for almost 69,37% of the total variance. The first factor, which accounted for 49.77% of the variance (eigenvalue = 3.48), is Cognitive resources (subcomponents: Self-efficacy, Self-esteem, Hope, Resilience, and Optimism). The second factor, which accounted for 19.60% of the variance (eigenvalue = 1.37), represents dimension Affective Reactivity (subcomponents: External Locus of Control and Neuroticism).

Table 3 presents scores on two factors extracted in factor analyses in the groups of the winter and summer hikers.

In last step, a discriminant function analysis (DFA) was used to assess the capacity of variables for the prediction of the winter and summer hikers. The variables for the group differences were included in the discriminant function analysis. The variables were Cognitive Resources and Affective Reactivity.

One significant function was identified, with an eigenvalue of 0.49 and canonical correlation of 0.57, F(2, 190) = 47.04, *p* < 0.01. Table 4 indicates that 77.20% of the group cases were correctly classified, this being 73.68% of the winter hikers and 80.61% of the summer hikers.

The Discriminant Function Analysis revealed that two factors contributed significantly to the multivariate discrimination between the mountain athletes. See Table 5.

## 4. Discussion

Previous research has rarely focused on the question of what personal resources help adventurers in the wildernesses to deal with demanding circumstances. The present aim is to examine personal resources among summer and winter hikers exploring the Tatra Mountains. Analysis showed that the personal resources distinguised winter mountain hikers from summer hikers. This finding supported the hypothesis.

Core Self-Evaluations differ between winter and summer hikers. The winter hikers had higher mean scores on Self-efficacy and Self-esteem, but lower mean scores on External Locus of Control and Neuroticism than summer hikers.

Higher scores on Self-efficacy and Self-esteem in the group of winter hikers indicate that they feel confident in their abilities. These beliefs can help them increase their efforts and persistence in the face of risk in natural environments or potential failure. It is probable that the above variables motivate hikers to engage in outdoor adventure. These results confirm previous studies related to self-efficacy in outdoor contexts [33].

The winter hikers control the events that influence their lives; thus, they perceive the mountain aspects of their risk to be at least partly controllable. They are more likely to take action to change the situation in a threatening environment when needed. Of course, we must remember that this isn’t actual, objective control of circumstances, but only a subjective feeling about controlling the external world. Subjective control can lead to underestimation of risks and, ultimately, can lead to accidents in the mountains [56,57].

Lower neuroticism in the winter hikers’ group positively relates to their internal locus of control. Winter hikers experience unpleasant emotions less often (e.g., anxiety or sadness) than controls. In this way, these emotions do not disturb their processing of information in stressful situations. This result confirms previous studies related to neuroticism in adventure contexts [36].

The winter hikers have higher Psychological Capital compared to summer hikers. This means that they have a tendency to look on the more favorable side of events in mountains, expect realization of their own goals, and perceive a capacity to find pathways to goals (higher optimism and hope). The result is both clear and understandable—without hope and optimism people will not even try to start a journey towards expressing one’s own needs, particularly if these needs are risky.

The winter hikers adapt better in the face of adversity, stress or threats compared to summer hikers (higher resilience). Moreover, if they experience some difficulties, they come back to life balance relatively faster than summer hikers. It seems that winter hikers have natural resources to battle against the power of nature.

The present aim is also to examine the structure of personal resources among winter and summer hikers. The results of factor analysis indicated two factors of personal resources. These factors distinguished the winter hikers from the summer hikers. The first factor was labelled Cognitive resources and it explains the highest percent of variance in psychological functioning of hikers in the context of personal resources. This means that this factor (from a psychological perspective) is most important for adaptation to wildernesses. In other worlds, cognitive processes are relatively more important in effective adaptation to the severe world of wild nature than control of negative emotions.

This factor includes the following variables: Self-efficacy, Self-esteem, Hope, Resiliency, and Optimism. It describes positive thinking about oneself, positive thinking about the future, or positive thinking about difficult situations in mountains. The winter hikers scored higher on this factor than to summer hikers. This means that they have a lot of cognitive resources at their disposal to cope with risks and to try to influence the outcomes of events, both positive and negative.

For them, the wilderness is probably a source of challenge and provides possibilities to express their own desires or goals. On the other hand, they probably perceive dangers in the wilderness as less risky because they strongly believe in their own competences or skills. Underestimating risk can lead to accidents in mountain environments.

These results suggest that winter hikers more often concentrate on endeavors to do something active to eliminate stressful circumstances. They try to change the nature of the stressor itself. Finally, they process information more effectively in extreme environments compared to summer hikers.

The second factor is Affective Reactivity. This factor includes the following subscales: Emotion Oriented Coping Avoidance, Oriented Coping, Locus of Control, and Neuroticism. It seems that this factor describes the emotional functioning of mountain athletes in dangerous, risky situations. A lower mean score on this factor in the winter hikers group suggests that this group can control stress more effectively than summer hikers. Winter hikers react with negative emotions (low neuroticism) significantly less often than summer hikers. This means that winter hikers do not panic in the face of inconvenience and experience less anxiety in dangerous environments compared to summer hikers. It is probable that winter hikers aren’t as “sensitive” to stress signals as summer hikers. They have subjective control of the wilderness; thus, their personal safety is reduced slower in risky situations than the recreationists preferring mountain trails in the summer.

Lower scores on Affective Reactivity in winter hikers also suggests that this group can manage negative emotions better that summer hikers; they do not panic in the wilderness because they can control stress. Moreover, lower scores on the Affective Reactivity factor in winter hikers suggests that they need help or support from others less often.

### Limitations of the Study and Future Directions

The first limitation of the study is the small number of participants. Another important limitation of the present study is that all of the respondents were young people. This fact limits the generalizability of the results. In future research, it will be important to assess not only young hikers, but other groups of adults.

In this study, the variable of gender was not controlled for. It seems that future research should also take into account the gender variable. Previous research suggests that variables of age and gender can play an important role in practicing outdoor recreation [58,59].

Under this study, only some of the variables of personal resources were subjected to analysis. Future research might encompass some of the concepts of personal resources which were not incorporated, such as, for example, temperamental traits, endurance, or briskness [60].

The current research focused only on mountain hikers. This means that the results can be applied to a very narrow population. Therefore, in future research, it would be interesting to compare personal resources of mountain hikers, recreationists in blue spaces (e.g., kayakers, sailors, and scuba divers), or air athletes (e.g., skydivers and paragliders).

Using comparative methodology would offer new data about adaptation to wild environment.

The data were collected in Poland. Several personal resources in this study concern specific local conditions, which are characteristic for Polish mountains. Hikers from different geographical regions may need other personal resources to explore mountains. An important limitation of the present study is that some differences between winter and summer hikers are small and should be considered more as a trend.

## 5. Conclusions

Research on wilderness exploration rarely focuses on the psychological traits important for adaptation in the wilderness. More often, researchers analyze the motivational aspects of outdoor adventure. Seeking new and stimulating experiences, the need for achievements, the need for freedom, or escaping the routine of life motivates people to undertake outdoor adventure. This perspective explains why the adventurous explore wildernesses, but it says little about the mechanisms of adaptation to the wild world of nature. The present research indicates that two components describe effective functioning in the wilderness: belief in one’s capabilities to explore severe surroundings, or perception dangers in the wilderness as less risky; and control of one’s fears in the wilderness. These components, with connecting motivation to explore wild nature, will allow us to understand exploration of extreme environment more fully.

## Figures and Tables

**Table 1:** Comparisons of Core Self-Evaluations and Psychological Capital in winter and summer hikers’ groups.

Personal Resources	Winter Hikers		Summer Hikers		t (191)	*p*	*Cohen’s d*
	M	SD	M	SD			
Core Self-Evaluations							
External locus of control	2.94	1.41	3.57	0.85	3.74	0.01	0.54
Neuroticism	4.43	1.20	5.26	0.96	5.28	0.01	0.76
Self-efficacy	3.32	0.45	2.79	0.44	8.15	0.01	1.19
Self-esteem	4.14	0.56	3.54	0.67	6.73	0.01	0.97
Psychological Capital							
Hope	3.34	0.49	2.81	0.49	7.37	0.01	1.08
Self-efficacy	3.32	0.45	2.79	0.44	8.15	0.01	1.19
Resilience	3.94	0.68	3.46	0.71	4.76	0.01	0.69
Optimism	3.04	0.58	2.64	0.58	4.66	0.01	0.68

**Table 2:** Exploratory Factor Analysis of the Core Self-Evaluations and Psychological Capital Scales.

	Variables	Factor 1	Factor 2
1	External Locus of Control		0.84
2	Neuroticism		0.79
3	Self-efficacy	0.88	
4	Self-esteem	0.83	
5	Hope	0.82	
6	Resilience	0.75	
7	Optimism	0.80	
	Explaining variance (%)	49.77	19.60
	Eigenvalue	3.48	1.37

**Table 3:** Comparisons of Personal Resources in the winter hikers and in the summer hikers.

Group						
Resources	Winter Hikers		Summer Hikers		t(191)	*p*
	M	SD	M	SD		
Cognitive resources	3.55	0.43	3.05	0.46	7.83	0.01
Affective Reactivity	3.69	1.09	4.42	0.71	5.49	0.01

**Table 4:** Classification results of the winter and the summer hikers.

		Predicted Group		Total
Cases		Winter Hikers *p* = 0.492	Summer Hikers *p* = 0.507	
Original Count	Winter hikers	70	25	95
	Summer hikers	80	18	98
% Classified	Winter hikers	73.68	26.32	100
	Summer hikers	80.61	19.39	100

Note: 77.20% of original grouped cases correctly classified.

**Table 5:** Summary of the discriminant function analysis.

Factors	Wilks’–Lambda	Partial–Lambda	F-Remove-(1.190)	*p*-Level	Toler.	1-Toler. (R-Sqr.)
Cognitive resources	0.86	0.77	55.26	0.01	0.99	0.01
Affective Reactivity	0.75	0.88	24.95	0.01	0.99	0.01

## Data Availability

The datasets used during this study are available from the corresponding author.

## References

[1] Nordbø I., Prebensen N.K. (2015). Hiking as mental and physical experience. Adv. Hosp. Leis..

[2] Kortenkamp K.V., Moore C.F., Sheridan D.P., Ahrens E.S. (2017). No hiking beyond this point! Hiking risk prevention recommendations in peer-reviewed literature. J. Outdoor Recreat. Tour..

[3] Próchniak P., Próchniak A. (2021). Future-Oriented Coping with Weather Stress among Mountain Hikers: Temperamental Personality Predictors and Profiles. Behav. Sci..

[4] Chhetri P., Arrowsmith C., Jackson M. (2004). Determining hiking experiences in nature-based tourist destinations. Tour. Manag..

[5] Rodrigues A., Kastenholz E., Rodrigues A. (2010). Hiking as a wellness activity. An exploratory study of hiking tourists in Portugal. J. Vacat. Mark..

[6] Niedermeier M., Einwanger J., Hartl A., Kopp M. (2017). Affective responses in mountain hiking-A randomized crossover trial focusing on differences between indoor and outdoor activity. PLoS ONE.

[7] Ochtyra A. (2020). Forest Disturbances in Polish Tatra Mountains for 1985–2016 in Relation to Topography, Stand Features, and Protection Zone. Forests.

[8] Smieja A. (2014). Flora of springs in the Polish Tatra Mountains—Habitat and phytosociological characteristics of crenophiles. Biodivers. Res. Conserv..

[9] Głowaciński Z., Makomaska-Juchiewicz M. (1992). Fauna of the Polish Tatra Mountains. Mt. Res. Dev..

[10] Ustrnul Z., Wypych A., Henek E., Czekierda D., Walawender J., Kubacka D., Pyrc R., Czernecki B. (2014). Meteorlogical Hazard Atlas of Poland.

[11] Próchniak P. (2020). Coping with Stress and Pain among Hard and Soft Adventure Mountain Athletes. Ann. Psychol..

[12] Swarbrooke J., Beard C., Leckie S., Pomfret G. (2003). Adventure Tourism: The New Frontier.

[13] Deroche T., Woodman T., Stephan Y., Brewer B., Le Scanff C. (2011). Athletes’ inclination to play through pain: A coping perspective. Anxiety Stress Coping.

[14] Schöffl V., Morrison A.B., Schwarz U., Schöffl I., Küpper T. (2010). Evaluation of injury and fatality risk in rock and ice climbing. Sports Medicin..

[15] Hobfoll S.E. (1989). Conservation of resources: A new attempt at conceptualizing stress. Am. Psychol..

[16] Pearlin L.I., Schooler C. (1978). The structure of coping. J. Health Soc. Behav..

[17] Hobfoll S.E. (1998). Stress, Culture, and Community.

[18] Hobfoll S.E., Folkman S. (2011). Conservation of resources theory: Its implication for stress, health, and resilience. The Oxford Handbook of Stress, Health, and Coping.

[19] Xanthopoulou D., Bakker A.B., Demerouti E., Schaufeli W.B. (2007). The role of personal resources in the job demands-resources model. Int. J. Stress Manag..

[20] Judge T.A., Locke E.A., Durham C.C. (1997). The dispositional causes of job satisfaction: A core evaluations approach. Res. Organ. Behav..

[21] Luthans F., Youssef C.M., Avolio B.J. (2007). Psychological Capital.

[22] Kerr J.H., Mackenzie S.H. (2013). Multiple motives for participating in adventure sports. Psychol. Sport Exerc..

[23] Woodman T., Hardy L., Barlow M., Le Scanff C. (2010). Motives for participation in prolonged engagement high-risk sports: An agentic emotion regulation perspective. Psychol. Sport Exerc..

[24] Brymer E., Schweitzer R. (2013). The search for freedom in extreme sports: A phenomenological exploration. Psychol. Sport Exerc..

[25] Brymer E., Schweizter R.D. (2017). Evoking the ineffable: The phenomenology of extreme Sports. Psychol. Conscious. Theory Res. Pract..

[26] Jones C.D., Hollenhorst S.J., Perna F., Selin S. (2000). Validation of The flow theory in an on-site whitewater kayaking setting. J. Leis. Res..

[27] Diehm A., Armatas C. (2004). Surfing: An avenue for socially acceptable risk-taking, satisfying needs for sensation seeking and experience seeking. Personal. Individ. Differ..

[28] Goma-i-Freixanet M. (1991). Personality profiles of subjects engaged in high physical risk sports. Personal. Individ. Differ..

[29] Slanger E., Rudestam K. (1997). Motivation and dishibition in high risk sports: Sensation seeking and self efficacy. J. Res. Personal..

[30] McCrae R.R., Costa P.T., Pervin L.A., John O.P. (1999). A Five-Factor theory of personality. Handbook of Personality: Theory and Research.

[31] Bandura A. (1997). Self-Efficacy: The Exercise of Control.

[32] Harter S. (1999). The Construction of the Self: A Developmental Perspective.

[33] Llewellyn D.J., Sanchez X. (2008). Individual differences and risk taking in rock climbing. Psychol. Sport Exerc..

[34] Egan S., Stelmack M.R. (2003). A personality profile of Mount Everest climbers. Personal. Individ. Differ..

[35] Levenson M.R. (1990). Risk taking and personality. J. Personal. Soc. Psychol..

[36] Tok S. (2011). The Big Five personality traits and risky sport participation. Soc. Behav. Personal. Int. J..

[37] Bahaeloo-Horeh S., Assari S. (2008). Students experience self-esteem improvement during mountaineering. Wilderness Environ. Med..

[38] Snyder C.R. (2005). Hope and the meaningful life: Theoretical and empirical associations between goal directed thinking and life-meaning. J. Soc. Clin. Psychol..

[39] Bandura A. (2004). Health Promotion by Social-Cognitive Means. Health Educ. Behav..

[40] Bonanno G.A. (2005). Resilience in the face of potential trauma. Curr. Dir. Psychol. Sci..

[41] Carver C.S., Scheier M.F., Aspinwall L.G., Staudinger U.M. (2002). Three Human Strengths. A Psychology of Human Strengths, Fundamental Questions and Future Directions for a Positive Psychology.

[42] Tukaiev S., Dolgova O., Ruzhenkova A., Lysenko O., Fedorchuk S., Ivaskevych D., Shynkaruk O., Denysova L., Usychenko V., Iakovenko O. (2020). Individual psychological determinants of stress resistance in rock climbers. J. Phys. Educ. Sport JPES.

[43] Harrison D., Sarkar M., Saward C., Sunderland C. (2021). Exploration of Psychological Resilience during a 25-Day Endurance Challenge in an Extreme Environment. Int. J. Environ. Res. Public Health.

[44] Drwal R.Ł. (1995). Adaptacja Kwestionariuszy Osobowości.

[45] Zawadzki B., Strelau J., Szczepaniak P., Śliwińska M. (1998). Inwentarz Osobowości NEO-FFI Costy i McCrae: Adaptacja polska. Podręcznik.

[46] Schwarzer R. (1998). General Perceived Self-Efficacy in 14 Cultures.

[47] Juczyński Z. (2000). Poczucie własnej skuteczności–Teoria i pomiar. Acta Univ. Lodziensis. Acta Psychol..

[48] Rosenberg M. (1965). Society and Adolescent Self-Image.

[49] Dzwonkowska I. (2008). , Lachowicz-Tabaczek, K., Łaguna, M SES. Polska Adaptacja Skali SES M. Rosenberga.

[50] Snyder C.R. (1995). Conceptualizing, measuring, & nurturing hope. J. Couns. Dev..

[51] Łaguna M., Trzebiński J., Zięba M. (2005). Kwestionariusz Nadziei na Sukces. Podręcznik.

[52] Block J., Kremen A.M. (1996). IQ and ego-resiliency: Conceptual and empirical connections and separateness. J. Personal. Soc. Psychol..

[53] Kaczmarek Ł. (2011). Kwestionariusz Sprężystości Psychicznej—Polska adaptacja Ego. Resiliency Scale. Czas. Psychol..

[54] Cudeck R., Tinsley H.E.A., Brown S.D. (2000). Exploratory factor analysis. Handbook of Applied Multivariate Statistics and Mathematical Modeling.

[55] Ledesma R.D., Valero-Mora P. (2007). Determining the number of factors to retain in EFA: An easy-to-use computer program for carrying out Parallel Analysis. Pract. Assess..

[56] Trimpop M. (1994). The Psychology of Risk Taking Behavior.

[57] Zuckerman M. (1994). Behavioral Expressions and Biosocial Bases of Sensation Seeking.

[58] Demirhan G. (2005). Mountaineers’ Risk Perception in Outdoor-Adventure Sports: A Study of Sex and Sports Experience. Percept. Mot. Ski..

[59] O’Connell T.S. (2010). The effects of age, gender and level of experience on motivation sea kayak. J. Adventure Educ. Outdoor Learn..

[60] Strelau J. (2013). Temperament. A Psychological Perspective.

